# A cross sectional study on Dutch layer farms to investigate the prevalence and potential risk factors for different *Chlamydia* species

**DOI:** 10.1371/journal.pone.0190774

**Published:** 2018-01-11

**Authors:** Marloes Heijne, Jeanet A. van der Goot, Helmi Fijten, Joke W. van der Giessen, Eric Kuijt, Catharina B. M. Maassen, Annika van Roon, Ben Wit, Ad P. Koets, Hendrik I. J. Roest

**Affiliations:** 1 Department of Bacteriology and Epidemiology, Wageningen Bioveterinary Research, Lelystad, the Netherlands; 2 Department of Infection Biology, Wageningen Bioveterinary Research, Lelystad, the Netherlands; 3 Center for Zoonoses and Environmental Microbiology, National Institute for Public Health and the Environment (RIVM), Bilthoven, the Netherlands; 4 Consumer and Safety Division, Food and Consumer Product Safety Authority (NVWA), Utrecht, the Netherlands; 5 Department of Farm Animal Health, Faculty of Veterinary Medicine, Utrecht University, Utrecht, the Netherlands; UAMS/ACHRI/ACNC, UNITED STATES

## Abstract

In poultry several *Chlamydia* species have been detected, but *Chlamydia psittaci* and *Chlamydia gallinacea* appear to be most prevalent and important. *Chlamydia psittaci* is a well-known zoonosis and is considered to be a pathogen of poultry. *Chlamydia gallinacea* has been described more recently. Its avian pathogenicity and zoonotic potential have to be further elucidated. Within the Netherlands no data were available on the presence of *Chlamydia* on poultry farms. As part of a surveillance programme for zoonotic pathogens in farm animals, we investigated pooled faecal samples from 151 randomly selected layer farms. On a voluntary base, 69 farmers, family members or farm workers from these 151 farms submitted a throat swab. All samples were tested with a generic 23S *Chlamydiaceae* PCR followed by a species specific PCR for *C*. *avium*, *C*. *gallinacea* and *C*. *psittaci*. *C*. *avium and psittaci* DNA was not detected at any of the farms. At 71 farms the positive result could be confirmed as *C*. *gallinacea*. Variables significantly associated with the presence of *C*. *gallinacea* in a final multivariable model were ‘age of hens,’ ‘use of bedding material’ and ‘the presence of horses.’ The presence of *C*. *gallinacea* was associated with neither clinical signs, varying from respiratory symptoms, nasal and ocular discharges to diarrhoea, nor with a higher mortality rate the day before the visit. All throat swabs from farmers, family members or farm workers tested negative for *Chlamydia* DNA, giving no further indication for possible bird-to-human (or human-to-bird) transmission.

## Introduction

*Chlamydia avium*, *Chlamydia gallinacea* and *Chlamydia psittaci* belong to the family of *Chlamydiaceae*, a group of obligate intracellular bacteria. *Chlamyia psittaci* is widespread and can infect over 465 bird species and several mammalian species, including humans [1]. Pathogenicity in animals depends on host species and *C*. *psittaci* strain. Clinical symptoms in birds vary from asymptomatic to acute death. *Chlamydia psittaci* is a well-known zoonosis and the cause of psittacosis. Transmission from birds to humans occurs via aerosolised respiratory or faecal excretions. In the Netherlands, psittacosis is notifiable in humans and pet birds but not in poultry. In poultry, chickens appeared to be less sensitive to chlamydial infection and a sporadic source of human infection [1–3]. However, in recent publications *C*. *psittaci* is regularly detected and chicken-to-human transmission is more frequently described [4–6].

*Chlamydia avium* and *C*. *gallinacea* have been detected in pet birds and poultry since 2009, first being classified as “atypical” and in 2014 added as new members of the genus *Chlamydia* [7–9]. *Chlamydia avium* has been found in psittacines and pigeons, *C*. *gallinacea* in chickens, guinea fowl and turkeys [10]. Recent studies hypothesised *C*. *gallinacea* to be endemic in chickens causing only mild clinical signs such as reduced weight gain in broilers [11]. Its zoonotic potential was suggested, but conclusive evidence has not been presented yet [8].

The impact of transmission of zoonotic pathogens from farm animals to humans was highlighted by the Dutch Q fever outbreak (2007–2010). Due to this outbreak, studies were initiated to assess the public health risks of intensive farming in densely populated areas [12]. One of the findings was a higher incidence of human pneumonia cases in the direct proximity of poultry farms [13, 14]. The cause of this higher incidence was unknown. We therefore hypothesised that *C*. *psittaci* or *C*. *gallinacea* could play a role. However, no data were available on the presence of *C*. *psittaci* and *C gallinacea* on Dutch poultry farms.

We investigated 755 faecal samples from 151 layer farms for the presence of *Chlamydiacea* DNA. Per farm a questionnaire was completed to identify possible risk factors. To gather information on possible bird to human transmission, farmers, family members or farm workers were invited to participate on a voluntary basis in throat swab sampling.

## Materials and methods

### Sampling strategy

**Fig 1:**
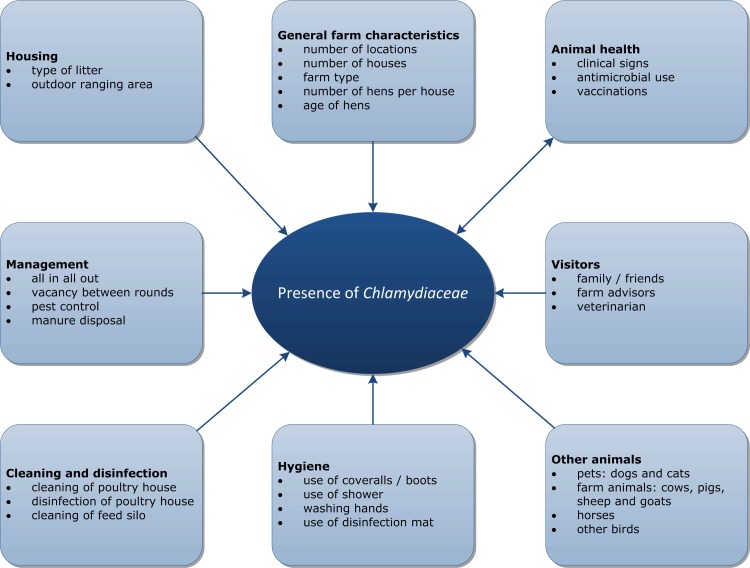
Overview of risk factors. Overview of possible risk factors for the presence of *Chlamydiaceae* on which information was gathered via a questionnaire.

Between March 2015 and January 2016, a cross-sectional study on layer farms was performed as part of a surveillance programme for zoonotic pathogens in farm animals. From the 993 layer farms in the Netherlands, 154 farms were randomly selected stratified on farming system (conventional n = 79, free range n = 34, organic n = 22, enriched cages n = 8, enriched colony n = 6). Finally, 151 farms completed a questionnaire and were included in the analysis. For *Chlamydia* testing, five pooled faecal samples were collected from one barn per farm, resulting in 755 samples. Each pooled sample contained twelve scoops of fresh faeces. Additional information on farm characteristics, husbandry practices, biosecurity measures, clinical history and antibiotic usage was acquired via a questionnaire (Fig 1). Farmers, family members and farm workers were asked to participate in the poultry-to-human transmission study by submitting two throat swabs (collected through self-sampling) for *Chlamydia* testing. In total 69 farmers, family members or farm workers from 41 farms participated in the study.

### Ethics statement

The Medical Ethics Review Committee (Utrecht Medical Centre, Utrecht) stated that the Medical Research Involving Human Subjects Act (WMO) does not apply to this study and therefore no official approval for the study is required under the WMO. All volunteers gave their written consent for participation in the study.

### Laboratory tests

DNA isolation of all pooled faecal samples was performed with a NucliSENS® easyMAG® (Biomerieux, Zaltbommel, the Netherlands). In brief, faecal material was taken from each sample with a dry swab, suspended in 1.5 ml Phosphate Buffered Saline (PBS) and thoroughly vortexed. From this suspension, 500 μl was added to 2 ml NucliSENS® lysis buffer for off-board lysis. After at least one hour of incubation at room temperature, the lysis buffer was added to 80 μl of silica and extracted according to manufacturer instructions for specific protocol B. Within this protocol an optimised washing protocol is used with extra and longer washing steps. The final elution volume was 100 μl. DNA isolation of human throat swabs was performed with a MagNA Pure® LC (Roche Diagnostics, Almere, The Netherlands) according to manufacturer instructions for off-board lysis. Of the sample 200 μl was processed to a final elution volume of 50 μl. *Chlamydiaceae*-DNA was detected using a generic PCR that targeted the 23S rRNA gene with primers and probes according to Ehricht et al [15]. *Chlamydia psittaci* DNA was detected using a PCR that targeted the *ompA* gene with primers and probes according to Pantchev et al [16]. For *C*. *avium* and *C*. *gallinacea* a duplex PCR was used targeting the *enoA* gene. For *C*. *avium* primer and probe sequences were used according to Zocevic et al [9]. For *C*. *gallinacea* primer and probe sequences were used according to Laroucau et al [6]. To validate the *C*. *avium* and *C*. *gallinacea* duplex PCR, 10-fold serial dilutions (single and in a mixture) of *C*. *gallinacea* strain 14DC0101 and *C*. *avium* strain 10DC97 were tested. The duplex PCR appeared to be as sensitive as the single PCR. No differences in Ct values were observed when *C*. *avium* and *C*. *gallinacea* were added in a single dilution or as a mixture. The final volume of the reaction mixture was 20 μl, including 5 μl of the DNA template, 10 μl TaqMan® Fast Universal PCR Master Mix (Applied Biosystems, Fisher Scientific, Landsmeer, the Netherlands), 1 μM of each primer, 0.2 μM of the probes, 0.2 μl UDG (5U/μl) and distilled PCR water to reach the final volume. Amplification was carried out in an ABI 7500 Fast Real-Time PCR system (Applied Biosystems, Fisher Scientific, Landsmeer, the Netherlands) using the following cycling parameters: 5 min at 37°C, 20 sec at 95°C, 50 cycles of 95°C for 3 sec and 60°C for 30 sec. As a control for DNA extraction, a known *C*. *psittaci* positive faecal swab was used. In each 23S and *C*. *psittaci* PCR run a dilution series of three *C*. *psittaci* DNA isolates was used as positive controls. In the *C*. *gallinacea* and *C*. *avium* duplex PCR, DNA from *C*. *gallinacea* strain 14DC0101 and *C*. *avium* 10DC97 and a mix of both strains were used as positive controls. Each real-time PCR run included a non-template control using 5 μl distilled water as template, and during the extraction per 12 samples a negative sample with 1.5 ml PBS was added. Samples with a Ct value up to 40 were considered positive and samples with a Ct value above 40 were considered negative. Farms were considered positive if at least one of five samples tested positive in the PCR.

### GIS map

**Fig 2:**
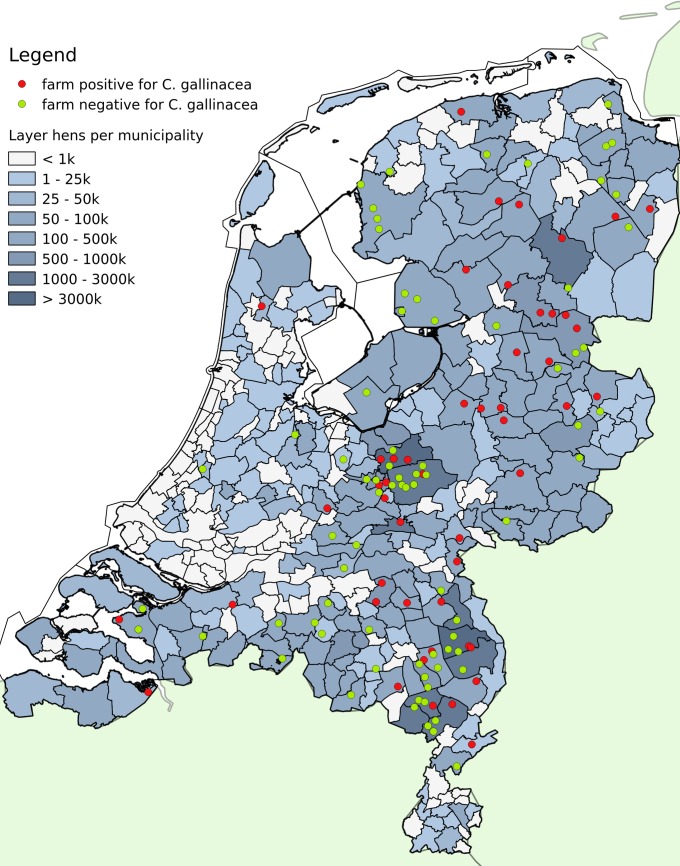
Map with *C*. *gallinacea* positive and negative farms. *Chlamydia gallinacea* positive and negative farms plotted on a laying hen density map of the Netherlands.

*Chlamydia gallinacea* positive and negative farms were plotted on a laying hen density map of the Netherlands (Fig 2). Data were extracted from CBS Statline (http://statline.cbs.nl) and imported into QGIS version 2.18.

### Statistical analyses

Farm prevalence was determined with an exact (Clopper-Pearson) 95 percent confidence interval (epitools.ausvet.com.au). Data from the questionnaires were collected via a digital form in Epi Info^TM^ and analysed using IBM SPSS Statistics for Windows, Version 23.0 (IBM Corp., Armonk, N.Y., USA). Overlapping variables or small categories were merged or summarised when possible. Potential risk factors for the presence of *C*. *gallinacea* were initially examined with a univariable analysis using a Chi square test or a logistic regression for continuous variables. Variables associated (*p* ≤ 0.20) with the outcome of interest (presence of *C*. *gallinacea*) were considered for inclusion in a stepwise, backward, multiple logistic regression analysis. The selected variables for the multivariable analysis were tested for mutual correlation. A likelihood ratio test was performed to eliminate variables from the multivariable model. Variables had to be significant (*p* ≤ 0.05) to remain in the final model. The goodness of fit of the final model was tested using the Hosmer and Lemeshow test.

## Results

*Chlamydiaceae* DNA was detected on 74 of the 151 farms and confirmed as *C*. *gallinacea* on 71 farms (farm prevalence 47%, 95% CI: 39–55%). Neither *C*. *psittaci* DNA nor *C*. *avium* DNA was detected in any of the samples from the 151 farms. The distribution of the number of positive samples per farm in the 23S *Chlamydiaceae* PCR and the *C*. *gallinacea* PCR is shown in Table 1. On 31 farms all five samples were positive in both the *Chlamydiaceae* PCR and *C*. *gallinacea* PCR, whereas on 67 farms all five samples were negative in both the *Chlamydiaceae* PCR and *C*. *gallinacea* PCR. At seven farms no *Chlamydiaceae* DNA was detected, but per farm one or two samples tested positive for C. *gallinacea* DNA with Ct values above 36. The 71 farms that had one or more positive samples in both the *Chlamydiaceae* PCR and the *C*. *gallinacea* PCR were included in the risk factor analysis.

**Table 1:** The distribution of the number of positive samples per farm in the *Chlamydiaceae* and *C*. *gallinacea* PCR.

		Number of positive samples per farm in *C*. *gallinacea* PCR (n = 5 per farm)						
		0	1	2	3	4	5	total
**Number of positive samples per farm in *Chlamydiaceae* PCR (n = 5 per farm)**	**0**	67	6	1	0	0	0	74
	**1**	2	6	1	1	0	0	10
	**2**	2	3	1	2	2	0	11
	**3**	0	2	1	3	0	1	7
	**4**	0	0	2	1	3	1	6
	**5**	0	0	0	1	3	30	34
	**total**	71	17	6	8	8	32	142*

* The results of 142 farms are shown. From seven farms one or more samples showed inhibition. From two farms only four samples could be tested. The results of these nine farms are not shown in the table, but the farm level results were used in the analysis.

The location of the positive and negative farms is shown in Fig 2. *Chlamydia gallinacea* positive and negative farms appear to be equally distributed in the Netherlands.

General descriptors about farm type and the median farm size are shown in Table 2. Farm type is related to farm size. Farms with enriched cages and colony systems are larger than free range and organic farms. Due to the relation with farm type, farm size was excluded from the analysis. Background and coding information on the variables in the univariable and multivariable analyses are added in the S1 and S2 Files. For the variables ‘age of hens’ and ‘manure disposal’ the smallest categories were merged. From the variable ‘vacancy period’ (period between two flocks, when the barn is empty), outliers with a vacancy period above 90 days were excluded from the analysis.

**Table 2:** General descriptors of farm type and farm size.

Farm type	Number of farms	% of participating farms	Median farm size (range)
Conventional (Barn egg)	79	52.3	33,696 (1,000–239,000)
Free range	34	22.8	24,410 (900–117,000)
Enriched cages	8	5.3	97,693 (648–180,000)
Enriched colony system	6	3.9	182,600 (66,000–383,000)
Organic	22	14.5	11850 (500–32,800)
Missing information	2	1.3	n.a.
Total	151	100	28,750 (500–383,000)

In the univariable analysis, ten variables met the criteria of *p* ≤ 0.2, i.e. ‘age of hens’, ‘use of bedding material’, ‘presence of horses’, ‘frequency of manure disposal’, ‘visitors have to shower before entrance’, ‘other birds’, ‘free range’, ‘vaccination against *Pasteurella multocida’* or ‘Egg Drop Syndrome’ and ‘vacancy period’ (Table 3). No mutual correlations were found between these ten variables and they were all included in the multivariable analysis. No associations were found between the presence of *C*. *gallinacea* and ‘one or more locations’, ‘more than one poultry house’, ‘all in all out at farm level’, ‘fly control’, ‘visitors’, ‘disinfection method’, ‘frequency of cleaning of the feed silo’, ‘washing hands before entrance’ and the ‘presence of other farm animals or pets’. All farms reported that they controlled rats and mice. The variables ‘use of disinfection mat before entrance’ and ‘use of tools in one or more houses’ were not included in the analysis, due to inconsistent answers in the questionnaires.

**Table 3:** Variables from the univariable analysis with *p* ≤ 0.2 (ranked by *p*-value).

Variable	No. of infected farms n = 71* (%)	No. of non-infected farms n = 80 (%)	Odds Ratio (CI 95%)	p-value (Chi square)
Age of hens^#^				< 0.01
*till 40 weeks*	*15/70 (21*.*4)*	*32/77 (41*.*6)*	*Ref*	*Ref*
*40–60 weeks*	*28/70 (40*.*0)*	*13/77 (16*.*9)*	*4*.*6 (1*.*87–11*.*29)*	*< 0*.*01*
*older than 60 weeks*	*27/70 (38*.*6)*	*32/77 (41*.*6)*	*1*.*80 (0*.*81–4*.*00)*	*0*.*15*
Use of bedding material	64/69 (92.8)	60/80 (75.0)	4.27 (1.51–12.09)	<0.01
Horses present	26/71 (36.6)	14/80 (17.5)	2.72 (1.28–5.78)	<0.01
Manure disposal^#^				0.08
*once or less than once a week*	*6/70 (8*.*6)*	*18/77 (23*.*4)*	*Ref*	*Ref*
*once every two weeks*	*21/70 (29*.*6)*	*14/77 (18*.*3)*	*4*.*50 (1*.*43–14*.*14)*	*0*.*10*
*once a month*	*17/70 (23*.*9)*	*18/77 (23*.*4)*	*2*.*83 (0*.*91–8*.*83)*	*0*.*07*
*less than once a month*	*26/70 (38*.*0)*	*27/77 (35*.*1)*	*8*.*89 (0*.*99–8*.*4)*	*0*.*05*
Use of shower before entrance (visitors)	6/71 (8.5)	14/80 (17.5)	0.44 (0.16–1.16)	0.10
Other birds present^i^	5/71 (7.0)	1/80 (1.3)	5.99 (0.68–52.5)	0.10
Vaccination against *Pasteurella multocida*^i^	8/71 (11.3)	3/80 (3.8)	3.26 (0.83–12.80)	0.12
Vacancy period^ii^			/	0.14
Vaccination against Egg Drop Syndrome	41/71 (57.7)	37/80 (46.3)	1.59 (0.83–3.02)	0.16
Free range sampled house	39/69 (42.0)	25/80 (31.3)	1.60 (0.81–3.12)	0.17

*Due to missing values, the number of farms per variable can differ

^#^p-value was calculated with logistic regression

^i^Fisher exact p-value was used (cells with counts n<5)

^ii^continuous variable

In the multivariable analysis, three variables were significantly associated with the presence of *C*. *gallinacea* as shown in Table 4: ‘age of hens’, ‘use of bedding material’ and ‘presence of horses’. The final model met the criteria of the Hosmer-Lemeshow goodness of fit test.

**Table 4:** Results of multivariable analysis (ranked by *p*-value).

Variable	Odds Ratio (CI 95%)	p-value
Age of hens		< 0.01
*till 40 weeks*	*ref*	*ref*
*40–60 weeks*	*5*.*41 (2*. *02–14*.*53)*	*< 0*.*01*
*older than 60 weeks*	*2*.*28 (0*.*94–5*.*53)*	*0*.*07*
Use of bedding material	4.22 (1.40–12.75)	0.01
Horses present	2.67 (1.16–6.12)	0.02

For the multivariable analysis 139 farms were selected, 12 had missing values for one or more of the selected variables.

No associations between the presence of *C*. *gallinacea* DNA and clinical signs, varying from respiratory symptoms and nasal and ocular discharges to diarrhoea, were found. The number of farms reporting clinical signs was low (n = 11). Also no association was found between the mortality rate the day before the visit and the presence of *C*. *gallinacea*. A total of 83 farms reported a mortality rate per day of < 0.01%, 58 a mortality rate between 0.01% and 0.05%, and 7 farms a mortality rate > 0.05% (3 farms did not report the mortality rate the day before the visit).

The 69 human throat swabs all tested negative in the *Chlamydiaceae* PCR. A total of 26 human samples were collected from farmers, family members or workers from 17 *C*. *gallinacea* DNA positive farms and 42 samples from 24 *C*. *gallinacea* DNA negative farms. One human sample could not be related to a sampled farm.

In summary, *C*. *gallinacea* DNA is highly prevalent on Dutch layer farms (farm prevalence 47%, 95% CI 39–55%), while neither *C*. *psittaci* DNA nor *C*. *avium* DNA were detected in any of the samples from the 151 farms. In the multivariable model, the presence of *C*. *gallinacea* appears to be associated with the ‘ age of hens’, ‘presence of horses’ and ‘use of bedding material’. No association was found with clinical signs or mortality rate the day before the visit. All of the 69 human throat swabs collected from farmers, family members or workers tested negative for *Chlamydiaceae* DNA.

## Discussion

Our cross-sectional study shows that *C*. *gallinacea* DNA is present on 47% (95% CI 39–55%) of layer farms in the Netherlands. The high prevalence of *C*. *gallinacea* DNA is in agreement with publications that postulate *C*. *gallinacea* to be the most important *Chlamydia* spp in chickens [11, 17]. In 2012, Zocevic et al. detected mainly DNA of atypical *Chlamydias* (later redefined as *C*. *gallinacea*) in 95 of 283 samples from different poultry flocks from France, Greece, Slovenia, Croatia and China [9]. Guo et al. detected *C*. *gallinacea* DNA in about 20% (359/1791) of oral and cloacal swabs of chickens from different provinces in China [11]. Hulin et al. reported a predominance of *C*. *gallinacea* in a poultry slaughterhouse where mainly chickens were slaughtered; in 52 / 129 flocks one or more samples were PCR positive for *C*. *gallinacea*.

*C*. *psittaci* and *C*. *avium* DNA were not detected at any of the 151 farms (95% CI 0–2%). These results are in line with the findings of Guo et al., where 41 of 1791 (2.3%) chicken samples were PCR positive for *C*. *psittaci* [11], and with the study of Hulin et al. where only one of the 129 flocks (bird species not specified) from the chicken slaughterhouse was PCR positive [17]. In contrast, Lagae et al. PCR detected and cultured *C*. *psittaci* from individual pharynx swabs from 7/7 broiler, 5/5 layer and 6/7 broiler breeder farms in Belgium. Differences in sampling methods might play a role. It has been shown that pharyngeal swabs are a more sensitive sampling method than cloacal swabs or faecal samples for the detection of *C*. *psittaci* [18]. Culturing, however has proven to be a less sensitive detection method than PCR, so this might not fully explain the large difference in prevalence [19]. The prevalence of *C*. *psittaci* might differ between countries. The absence of *C*. *avium* was expected. So far, this bacterium has only been found in psittacines and pigeons and not in poultry [7].

In the risk factor analysis ‘ age of hens’, ‘use of bedding material’ and ‘presence of horses’ were associated with the presence of *C*. *gallinacea*. The age related risk for the presence of *C*. *gallinacea* peaks between 40 and 60 weeks (OR 5.41, *p* < 0.01). Factors that might influence this risk are the moment of introduction, the duration of *C*. *gallinacea* infections and the acquisition of immunity. However, this information is currently not available for *C*. *gallinacea* infections. Studies with a more longitudinal approach are therefore needed. The association with the ‘use of bedding material’ might be explained by the introduction of the bacterium via bedding material or the effect of this material on the persistence of the bacterium in the environment. It has been reported that the elementary bodies of other *Chlamydiaceae* can survive in litter for several months [20]. There is no obvious explanation for the association with the ‘presence of horses’. Several *Chlamydia* species have been detected in horses, but the presence of *C*. *gallinacea* has not been described [21]. However, *C*. *gallinacea* has been detected in vaginal swabs from cattle in China suggesting it might not be restricted to poultry [22]. There might be other associated factors as well, such as frequent movement of trailers, which explains the association with horses. More detailed studies are needed to confirm the relation between the risk factors in the final model and the presence of *C*. *gallinacea* DNA.

We did not observe an association between the presence of *C*. *gallinacea* and ‘clinical signs’, based on the results of the questionnaire. It should be noted that only 11 farms reported overt clinical problems, which varied from respiratory symptoms and nasal and ocular discharges to diarrhoea. Also no association was found with the mortality rate the day before the visit. An association with increased mortality cannot be excluded, because the mortality rate might have increased earlier in the infection and subsequently returned to a normal level. To study this we should have analysed for a period longer than 1 day before the visit. Furthermore, a possible clinical outcome of a *C*. *gallinacea* infection could be more subtle or subclinical. For example Guo et al. did not report any clinical signs, but did find a reduction in growth of broiler chicks [11]. Reinhold et al. discussed the role of *Chlamydiaceae* in cattle and suggested subclinical and chronic chlamydial infections might be economically more important than a clinical outbreak [20]. Further studies should also take into account subclinical or more economically important parameters, such as egg production during the entire production round.

All human samples collected tested negative for *Chlamydiacea* DNA. Participants were not selected for clinical signs and 26 were working or living at a *C*. *gallinacea* positive farm. A positive sample would have given an indication of possible bird-to-human (or human-to-bird) transmission. To date *C*. *gallinacea* has only been suggested as a cause of human pneumonia [8], but in our study we could not confirm this. Sputum or bronchoalveolar lavage fluid (BAL) from patients with community acquired pneumonia (CAP) should be examined to further investigate whether *C*. *gallinacea* could be a cause of human pneumonia.

Our study adds to the hypothesis that *C*. *gallinacea* is the endemic *Chlamydia* of chickens. However, many questions still need to be answered. The most important of these is to elucidate the zoonotic potential of C. *gallinacea* and to investigate the pathogenesis of a *C*. *gallinacea* infection, as these could be of economic significance for the poultry sector.

## Supporting information

S1 File Background data risk factor analysis. This file holds data on the variables used in the univariable and multivariable analysis.
(XLSX)

S2 File Variable coding information. This file holds information on the coding of the variables.
(HTM)
