# Supplementary material for: A cross sectional study on Dutch layer farms to investigate the prevalence and potential risk factors for different Chlamydia species
Source: PLoS One. 2018 Jan 11;13(1):e0190774. doi: 10.1371/journal.pone.0190774 (PMC5764275; doi:10.1371/journal.pone.0190774)
Supplement: S2 File — This file holds information on the coding of the variables. (HTM) [file pone.0190774.s002.htm]

xml version="1.0" encoding="UTF-8"?


S2\_20170723\_codebookvariables


|  |  |  |
| --- | --- | --- |
| IBM SPSS Web Report - Output3     ---   Contents  Previous  Next  Help |  | Controls disabled by the system     --- |

- Codebook

  - File Information
  - ResultFL
  - Result23S
  - Ppools23S
  - ResultCgal
  - PpoolsCgal
  - ResultCpsi
  - NumberHouses
  - NumberLocations
  - Numberhens
  - FreeRange
  - Freerange\_inv
  - FarmAnimals
  - Horses
  - Horses\_inv
  - Pets
  - Birds
  - Birds\_inv
  - Age\_catsum
  - Age\_catsum2
  - Allinallout
  - Pestcontrol
  - Flycontrol
  - Visitvet
  - Visitfamily
  - Visitfriends
  - Visitadvisor
  - Litter
  - Litter\_inverse
  - Cleaning\_2cat
  - Disinfection
  - Cleaningsilo\_catsum
  - Vacancy\_exoutliers
  - Manuredisposal\_catsum
  - WasHand\_catsum
  - Shower\_sum
  - Shower\_inverse
  - Clinsigns
  - TreatmentAB
  - Mortality
  - Coccidiosis
  - Gumborovac
  - PMvac
  - PMvac\_inv
  - IBvac
  - ILTvac
  - TRTofAMPVvac
  - SalEntvac
  - SalTyphvak
  - Marekvac
  - AEvac
  - Mgvac
  - Msvac
  - Erysipelasvac
  - PDvac
  - EDSvac
  - EDSvac\_inv
  - ZRE\_1
  - DFB0\_1
  - DFB1\_1
  - DFB2\_1
  - DFB3\_1
  - DFB4\_1
  - COO\_1
  - LEV\_1

- Delete

Codebook  
Codebook - File Information - July 23, 2017

File InformationFile Information, table, 0 levels of column headers and 1 levels of row headers, table with 2 columns and 2 rows

|  |

Codebook  
Codebook - ResultFL - July 23, 2017

ResultFLResultFL, table, 1 levels of column headers and 2 levels of row headers, table with 3 columns and 7 rows

|  |  |  |
| --- | --- | --- |
|  | | Value |
| Standard Attributes | Position | 3 |
|  | Label | Result PCRs at farm level |
|  | Type | Numeric |
| Valid Values | 0 | negative |
|  | 1 | positive |
|  |  |  |

Codebook  
Codebook - Result23S - July 23, 2017

Result23SResult23S, table, 1 levels of column headers and 2 levels of row headers, table with 3 columns and 7 rows

|  |  |  |
| --- | --- | --- |
|  | | Value |
| Standard Attributes | Position | 4 |
|  | Label | Result 23S Chlamydiacea PCR |
|  | Type | Numeric |
| Valid Values | 0 | negative |
|  | 1 | positive |
|  |  |  |

Codebook  
Codebook - Ppools23S - July 23, 2017

Ppools23SPpools23S, table, 1 levels of column headers and 2 levels of row headers, table with 3 columns and 11 rows

|  |  |  |
| --- | --- | --- |
|  | | Value |
| Standard Attributes | Position | 5 |
|  | Label | Number positive pools Chlamydiaceae PCR |
|  | Type | Numeric |
| Valid Values | 0 | 0 |
|  | 1 | 1 |
|  | 2 | 2 |
|  | 3 | 3 |
|  | 4 | 4 |
|  | 5 | 5 |
|  |  |  |

Codebook  
Codebook - ResultCgal - July 23, 2017

ResultCgalResultCgal, table, 1 levels of column headers and 2 levels of row headers, table with 3 columns and 7 rows

|  |  |  |
| --- | --- | --- |
|  | | Value |
| Standard Attributes | Position | 6 |
|  | Label | Result of C. gallinacea PCR |
|  | Type | Numeric |
| Valid Values | 0 | negative |
|  | 1 | positive |
|  |  |  |

Codebook  
Codebook - PpoolsCgal - July 23, 2017

PpoolsCgalPpoolsCgal, table, 1 levels of column headers and 2 levels of row headers, table with 3 columns and 11 rows

|  |  |  |
| --- | --- | --- |
|  | | Value |
| Standard Attributes | Position | 7 |
|  | Label | Number positive pools C. gallinacea PCR |
|  | Type | Numeric |
| Valid Values | 0 | 0 |
|  | 1 | 1 |
|  | 2 | 2 |
|  | 3 | 3 |
|  | 4 | 4 |
|  | 5 | 5 |
|  |  |  |

Codebook  
Codebook - ResultCpsi - July 23, 2017

ResultCpsiResultCpsi, table, 1 levels of column headers and 2 levels of row headers, table with 3 columns and 5 rows

|  |  |  |
| --- | --- | --- |
|  | | Value |
| Standard Attributes | Position | 8 |
|  | Label | Result of Chlamydia psittaci PCR |
|  | Type | Numeric |
|  |  |  |

Codebook  
Codebook - NumberHouses - July 23, 2017

NumberHousesNumberHouses, table, 1 levels of column headers and 2 levels of row headers, table with 3 columns and 7 rows

|  |  |  |
| --- | --- | --- |
|  | | Value |
| Standard Attributes | Position | 9 |
|  | Label | Number of houses |
|  | Type | Numeric |
| Valid Values | 0 | one house |
|  | 1 | more than one house |
|  |  |  |

Codebook  
Codebook - NumberLocations - July 23, 2017

NumberLocationsNumberLocations, table, 1 levels of column headers and 2 levels of row headers, table with 3 columns and 7 rows

|  |  |  |
| --- | --- | --- |
|  | | Value |
| Standard Attributes | Position | 10 |
|  | Label | Number of locations |
|  | Type | Numeric |
| Valid Values | 0 | one location |
|  | 1 | more than one location |
|  |  |  |

Codebook  
Codebook - Numberhens - July 23, 2017

NumberhensNumberhens, table, 1 levels of column headers and 2 levels of row headers, table with 3 columns and 5 rows

|  |  |  |
| --- | --- | --- |
|  | | Value |
| Standard Attributes | Position | 11 |
|  | Label | Number of layer hens in sampled house |
|  | Type | Numeric |
|  |  |  |

Codebook  
Codebook - FreeRange - July 23, 2017

FreeRangeFreeRange, table, 1 levels of column headers and 2 levels of row headers, table with 3 columns and 7 rows

|  |  |  |
| --- | --- | --- |
|  | | Value |
| Standard Attributes | Position | 12 |
|  | Label | Free range sampled house |
|  | Type | Numeric |
| Valid Values | 0 | no |
|  | 0 | yes |
|  |  |  |

Codebook  
Codebook - Freerange\_inv - July 23, 2017

Freerange\_invFreerange\_inv, table, 1 levels of column headers and 2 levels of row headers, table with 3 columns and 7 rows

|  |  |  |
| --- | --- | --- |
|  | | Value |
| Standard Attributes | Position | 13 |
|  | Label | Free range inverse |
|  | Type | Numeric |
| Valid Values | 0 | yes |
|  | 1 | no |
|  |  |  |

Codebook  
Codebook - FarmAnimals - July 23, 2017

FarmAnimalsFarmAnimals, table, 1 levels of column headers and 2 levels of row headers, table with 3 columns and 7 rows

|  |  |  |
| --- | --- | --- |
|  | | Value |
| Standard Attributes | Position | 14 |
|  | Label | Dairy, pigs, calves, sheeps or goats present |
|  | Type | Numeric |
| Valid Values | 0 | no other farm animals |
|  | 1 | other farm animals present |
|  |  |  |

Codebook  
Codebook - Horses - July 23, 2017

HorsesHorses, table, 1 levels of column headers and 2 levels of row headers, table with 3 columns and 7 rows

|  |  |  |
| --- | --- | --- |
|  | | Value |
| Standard Attributes | Position | 15 |
|  | Label | Horses present |
|  | Type | Numeric |
| Valid Values | 0 | no |
|  | 1 | yes |
|  |  |  |

Codebook  
Codebook - Horses\_inv - July 23, 2017

Horses\_invHorses\_inv, table, 1 levels of column headers and 2 levels of row headers, table with 3 columns and 7 rows

|  |  |  |
| --- | --- | --- |
|  | | Value |
| Standard Attributes | Position | 16 |
|  | Label | Horses present inverse |
|  | Type | Numeric |
| Valid Values | 0 | yes |
|  | 1 | no |
|  |  |  |

Codebook  
Codebook - Pets - July 23, 2017

PetsPets, table, 1 levels of column headers and 2 levels of row headers, table with 3 columns and 7 rows

|  |  |  |
| --- | --- | --- |
|  | | Value |
| Standard Attributes | Position | 17 |
|  | Label | Dogs or cats present |
|  | Type | Numeric |
| Valid Values | 0 | not present |
|  | 1 | present |
|  |  |  |

Codebook  
Codebook - Birds - July 23, 2017

BirdsBirds, table, 1 levels of column headers and 2 levels of row headers, table with 3 columns and 7 rows

|  |  |  |
| --- | --- | --- |
|  | | Value |
| Standard Attributes | Position | 18 |
|  | Label | Other birds present |
|  | Type | Numeric |
| Valid Values | 0 | no |
|  | 1 | yes |
|  |  |  |

Codebook  
Codebook - Birds\_inv - July 23, 2017

Birds\_invBirds\_inv, table, 1 levels of column headers and 2 levels of row headers, table with 3 columns and 7 rows

|  |  |  |
| --- | --- | --- |
|  | | Value |
| Standard Attributes | Position | 19 |
|  | Label | Birds inverse |
|  | Type | Numeric |
| Valid Values | 0 | present |
|  | 1 | not present |
|  |  |  |

Codebook  
Codebook - Age\_catsum - July 23, 2017

Age\_catsumAge\_catsum, table, 1 levels of column headers and 2 levels of row headers, table with 3 columns and 9 rows

|  |  |  |
| --- | --- | --- |
|  | | Value |
| Standard Attributes | Position | 20 |
|  | Label | Age in sampled house categories summarized |
|  | Type | Numeric |
| Valid Values | 2 | till 40 weeks |
|  | 3 | 40-60 weeks |
|  | 4 | 60-80 weeks |
|  | 5 | older than 80 weeks |
|  |  |  |

Codebook  
Codebook - Age\_catsum2 - July 23, 2017

Age\_catsum2Age\_catsum2, table, 1 levels of column headers and 2 levels of row headers, table with 3 columns and 8 rows

|  |  |  |
| --- | --- | --- |
|  | | Value |
| Standard Attributes | Position | 21 |
|  | Label | Age in sampled house categories summarized |
|  | Type | Numeric |
| Valid Values | 0 | < 40 weeks |
|  | 1 | 40-60 weeks |
|  | 2 | > 60 weeks |
|  |  |  |

Codebook  
Codebook - Allinallout - July 23, 2017

AllinalloutAllinallout, table, 1 levels of column headers and 2 levels of row headers, table with 3 columns and 7 rows

|  |  |  |
| --- | --- | --- |
|  | | Value |
| Standard Attributes | Position | 22 |
|  | Label | All in all out farm level |
|  | Type | Numeric |
| Valid Values | 0 | no |
|  | 1 | yes |
|  |  |  |

Codebook  
Codebook - Pestcontrol - July 23, 2017

PestcontrolPestcontrol, table, 1 levels of column headers and 2 levels of row headers, table with 3 columns and 7 rows

|  |  |  |
| --- | --- | --- |
|  | | Value |
| Standard Attributes | Position | 23 |
|  | Label | Control of mice and rats |
|  | Type | Numeric |
| Valid Values | 0 | no |
|  | 1 | yes |
|  |  |  |

Codebook  
Codebook - Flycontrol - July 23, 2017

FlycontrolFlycontrol, table, 1 levels of column headers and 2 levels of row headers, table with 3 columns and 7 rows

|  |  |  |
| --- | --- | --- |
|  | | Value |
| Standard Attributes | Position | 24 |
|  | Label | Fly control |
|  | Type | Numeric |
| Valid Values | 0 | no |
|  | 1 | yes |
|  |  |  |

Codebook  
Codebook - Visitvet - July 23, 2017

VisitvetVisitvet, table, 1 levels of column headers and 2 levels of row headers, table with 3 columns and 7 rows

|  |  |  |
| --- | --- | --- |
|  | | Value |
| Standard Attributes | Position | 25 |
|  | Label | Farm visitors: Veterinarian |
|  | Type | Numeric |
| Valid Values | 0 | no visits |
|  | 1 | visits |
|  |  |  |

Codebook  
Codebook - Visitfamily - July 23, 2017

VisitfamilyVisitfamily, table, 1 levels of column headers and 2 levels of row headers, table with 3 columns and 7 rows

|  |  |  |
| --- | --- | --- |
|  | | Value |
| Standard Attributes | Position | 26 |
|  | Label | Farm visitors: Family members |
|  | Type | Numeric |
| Valid Values | 0 | no |
|  | 1 | yes |
|  |  |  |

Codebook  
Codebook - Visitfriends - July 23, 2017

VisitfriendsVisitfriends, table, 1 levels of column headers and 2 levels of row headers, table with 3 columns and 7 rows

|  |  |  |
| --- | --- | --- |
|  | | Value |
| Standard Attributes | Position | 27 |
|  | Label | Farm visitors: Friends or family outside household |
|  | Type | String |
| Valid Values | 0 | no |
|  | 1 | yes |
|  |  |  |

Codebook  
Codebook - Visitadvisor - July 23, 2017

VisitadvisorVisitadvisor, table, 1 levels of column headers and 2 levels of row headers, table with 3 columns and 7 rows

|  |  |  |
| --- | --- | --- |
|  | | Value |
| Standard Attributes | Position | 28 |
|  | Label | Farm visitors: Advisor |
|  | Type | String |
| Valid Values | 0 | nee |
|  | 1 | yes |
|  |  |  |

Codebook  
Codebook - Litter - July 23, 2017

LitterLitter, table, 1 levels of column headers and 2 levels of row headers, table with 3 columns and 7 rows

|  |  |  |
| --- | --- | --- |
|  | | Value |
| Standard Attributes | Position | 29 |
|  | Label | Use of litter |
|  | Type | Numeric |
| Valid Values | 0 | no |
|  | 1 | yes |
|  |  |  |

Codebook  
Codebook - Litter\_inverse - July 23, 2017

Litter\_inverseLitter\_inverse, table, 1 levels of column headers and 2 levels of row headers, table with 3 columns and 7 rows

|  |  |  |
| --- | --- | --- |
|  | | Value |
| Standard Attributes | Position | 30 |
|  | Label | Use of litter inverse |
|  | Type | Numeric |
| Valid Values | 0 | yes |
|  | 1 | no |
|  |  |  |

Codebook  
Codebook - Cleaning\_2cat - July 23, 2017

Cleaning\_2catCleaning\_2cat, table, 1 levels of column headers and 2 levels of row headers, table with 3 columns and 7 rows

|  |  |  |
| --- | --- | --- |
|  | | Value |
| Standard Attributes | Position | 31 |
|  | Label | Cleaning wet or dry categories summarized |
|  | Type | Numeric |
| Valid Values | 0 | nat reinigen |
|  | 1 | alleen droog |
|  |  |  |

Codebook  
Codebook - Disinfection - July 23, 2017

DisinfectionDisinfection, table, 1 levels of column headers and 2 levels of row headers, table with 3 columns and 9 rows

|  |  |  |
| --- | --- | --- |
|  | | Value |
| Standard Attributes | Position | 32 |
|  | Label | Disinfection wet or fumigation |
|  | Type | String |
| Valid Values | 0 | nat desinfecteren |
|  | 1 | uitgassen |
|  | 2 | uitgassen en nat desinfecteren |
| Missing Values | 3 | anders |
|  |  |  |

Codebook  
Codebook - Cleaningsilo\_catsum - July 23, 2017

Cleaningsilo\_catsumCleaningsilo\_catsum, table, 1 levels of column headers and 2 levels of row headers, table with 3 columns and 8 rows

|  |  |  |
| --- | --- | --- |
|  | | Value |
| Standard Attributes | Position | 33 |
|  | Label | Cleaning silo categories summarized |
|  | Type | Numeric |
| Valid Values | 0 | every round |
|  | 1 | other |
|  | 2 | never |
|  |  |  |

Codebook  
Codebook - Vacancy\_exoutliers - July 23, 2017

Vacancy\_exoutliersVacancy\_exoutliers, table, 1 levels of column headers and 2 levels of row headers, table with 3 columns and 5 rows

|  |  |  |
| --- | --- | --- |
|  | | Value |
| Standard Attributes | Position | 34 |
|  | Label | Vacancy period excl outliers > 90 days |
|  | Type | Numeric |
|  |  |  |

Codebook  
Codebook - Manuredisposal\_catsum - July 23, 2017

Manuredisposal\_catsumManuredisposal\_catsum, table, 1 levels of column headers and 2 levels of row headers, table with 3 columns and 9 rows

|  |  |  |
| --- | --- | --- |
|  | | Value |
| Standard Attributes | Position | 35 |
|  | Label | Frequency manure disposal categories summarized |
|  | Type | Numeric |
| Valid Values | 1 | once or less than once a week |
|  | 2 | once every two weeks |
|  | 3 | once a month |
|  | 4 | less than once a month |
|  |  |  |

Codebook  
Codebook - WasHand\_catsum - July 23, 2017

WasHand\_catsumWasHand\_catsum, table, 1 levels of column headers and 2 levels of row headers, table with 3 columns and 8 rows

|  |  |  |
| --- | --- | --- |
|  | | Value |
| Standard Attributes | Position | 36 |
|  | Label | Washing hands categories summarized |
|  | Type | Numeric |
| Valid Values | 2 | soap |
|  | 3 | desinfection gel |
| Missing Values | 0 | not, sometimes or only water |
|  |  |  |

Codebook  
Codebook - Shower\_sum - July 23, 2017

Shower\_sumShower\_sum, table, 1 levels of column headers and 2 levels of row headers, table with 3 columns and 7 rows

|  |  |  |
| --- | --- | --- |
|  | | Value |
| Standard Attributes | Position | 37 |
|  | Label | Visitors shower before entrance |
|  | Type | Numeric |
| Valid Values | 0 | no |
|  | 1 | yes |
|  |  |  |

Codebook  
Codebook - Shower\_inverse - July 23, 2017

Shower\_inverseShower\_inverse, table, 1 levels of column headers and 2 levels of row headers, table with 3 columns and 7 rows

|  |  |  |
| --- | --- | --- |
|  | | Value |
| Standard Attributes | Position | 38 |
|  | Label | Visitors shower inverse |
|  | Type | Numeric |
| Valid Values | 0 | yes |
|  | 1 | no |
|  |  |  |

Codebook  
Codebook - Clinsigns - July 23, 2017

ClinsignsClinsigns, table, 1 levels of column headers and 2 levels of row headers, table with 3 columns and 7 rows

|  |  |  |
| --- | --- | --- |
|  | | Value |
| Standard Attributes | Position | 39 |
|  | Label | Clinical signs |
|  | Type | Numeric |
| Valid Values | 0 | no |
|  | 1 | yes |
|  |  |  |

Codebook  
Codebook - TreatmentAB - July 23, 2017

TreatmentABTreatmentAB, table, 1 levels of column headers and 2 levels of row headers, table with 3 columns and 7 rows

|  |  |  |
| --- | --- | --- |
|  | | Value |
| Standard Attributes | Position | 40 |
|  | Label | Treatment with antibiotics |
|  | Type | String |
| Valid Values | 0 | no |
|  | 1 | yes |
|  |  |  |

Codebook  
Codebook - Mortality - July 23, 2017

MortalityMortality, table, 1 levels of column headers and 2 levels of row headers, table with 3 columns and 7 rows

|  |  |  |
| --- | --- | --- |
|  | | Value |
| Standard Attributes | Position | 41 |
|  | Label | Mortality rate day before visit |
|  | Type | Numeric |
| Valid Values | 0 | Less than 0,01% (1 at 10000) |
|  | 1 | Above 0,01% (above 1 per 10000) |
|  |  |  |

Codebook  
Codebook - Coccidiosis - July 23, 2017

CoccidiosisCoccidiosis, table, 1 levels of column headers and 2 levels of row headers, table with 3 columns and 7 rows

|  |  |  |
| --- | --- | --- |
|  | | Value |
| Standard Attributes | Position | 42 |
|  | Label | Vaccination against coccidiosis |
|  | Type | Numeric |
| Valid Values | 0 | no |
|  | 1 | yes |
|  |  |  |

Codebook  
Codebook - Gumborovac - July 23, 2017

GumborovacGumborovac, table, 1 levels of column headers and 2 levels of row headers, table with 3 columns and 7 rows

|  |  |  |
| --- | --- | --- |
|  | | Value |
| Standard Attributes | Position | 43 |
|  | Label | Vaccination against Gumboro |
|  | Type | Numeric |
| Valid Values | 0 | no |
|  | 1 | yes |
|  |  |  |

Codebook  
Codebook - PMvac - July 23, 2017

PMvacPMvac, table, 1 levels of column headers and 2 levels of row headers, table with 3 columns and 7 rows

|  |  |  |
| --- | --- | --- |
|  | | Value |
| Standard Attributes | Position | 44 |
|  | Label | Vaccination against Pasteurella |
|  | Type | Numeric |
| Valid Values | 0 | no |
|  | 1 | yes |
|  |  |  |

Codebook  
Codebook - PMvac\_inv - July 23, 2017

PMvac\_invPMvac\_inv, table, 1 levels of column headers and 2 levels of row headers, table with 3 columns and 7 rows

|  |  |  |
| --- | --- | --- |
|  | | Value |
| Standard Attributes | Position | 45 |
|  | Label | Vaccination Pasteurella inverse |
|  | Type | Numeric |
| Valid Values | 0 | yes |
|  | 1 | no |
|  |  |  |

Codebook  
Codebook - IBvac - July 23, 2017

IBvacIBvac, table, 1 levels of column headers and 2 levels of row headers, table with 3 columns and 7 rows

|  |  |  |
| --- | --- | --- |
|  | | Value |
| Standard Attributes | Position | 46 |
|  | Label | Vaccination against IB |
|  | Type | Numeric |
| Valid Values | 0 | no |
|  | 1 | yes |
|  |  |  |

Codebook  
Codebook - ILTvac - July 23, 2017

ILTvacILTvac, table, 1 levels of column headers and 2 levels of row headers, table with 3 columns and 7 rows

|  |  |  |
| --- | --- | --- |
|  | | Value |
| Standard Attributes | Position | 47 |
|  | Label | Vaccination against ILT |
|  | Type | Numeric |
| Valid Values | 0 | no |
|  | 1 | yes |
|  |  |  |

Codebook  
Codebook - TRTofAMPVvac - July 23, 2017

TRTofAMPVvacTRTofAMPVvac, table, 1 levels of column headers and 2 levels of row headers, table with 3 columns and 7 rows

|  |  |  |
| --- | --- | --- |
|  | | Value |
| Standard Attributes | Position | 48 |
|  | Label | Vaccination against TRT / AMPV |
|  | Type | Numeric |
| Valid Values | 0 | no |
|  | 1 | yes |
|  |  |  |

Codebook  
Codebook - SalEntvac - July 23, 2017

SalEntvacSalEntvac, table, 1 levels of column headers and 2 levels of row headers, table with 3 columns and 7 rows

|  |  |  |
| --- | --- | --- |
|  | | Value |
| Standard Attributes | Position | 49 |
|  | Label | Vaccination against Salmonella enteritidis |
|  | Type | Numeric |
| Valid Values | 0 | no |
|  | 1 | yes |
|  |  |  |

Codebook  
Codebook - SalTyphvak - July 23, 2017

SalTyphvakSalTyphvak, table, 1 levels of column headers and 2 levels of row headers, table with 3 columns and 7 rows

|  |  |  |
| --- | --- | --- |
|  | | Value |
| Standard Attributes | Position | 50 |
|  | Label | Vaccination against Salmonella typhimurium |
|  | Type | Numeric |
| Valid Values | 0 | no |
|  | 1 | yes |
|  |  |  |

Codebook  
Codebook - Marekvac - July 23, 2017

MarekvacMarekvac, table, 1 levels of column headers and 2 levels of row headers, table with 3 columns and 7 rows

|  |  |  |
| --- | --- | --- |
|  | | Value |
| Standard Attributes | Position | 51 |
|  | Label | Vaccination against Marek |
|  | Type | Numeric |
| Valid Values | 0 | no |
|  | 1 | yes |
|  |  |  |

Codebook  
Codebook - AEvac - July 23, 2017

AEvacAEvac, table, 1 levels of column headers and 2 levels of row headers, table with 3 columns and 7 rows

|  |  |  |
| --- | --- | --- |
|  | | Value |
| Standard Attributes | Position | 52 |
|  | Label | Vaccination against AE |
|  | Type | Numeric |
| Valid Values | 0 | no |
|  | 1 | yes |
|  |  |  |

Codebook  
Codebook - Mgvac - July 23, 2017

MgvacMgvac, table, 1 levels of column headers and 2 levels of row headers, table with 3 columns and 7 rows

|  |  |  |
| --- | --- | --- |
|  | | Value |
| Standard Attributes | Position | 53 |
|  | Label | Vaccination against Mycoplasma gallisepticum |
|  | Type | Numeric |
| Valid Values | 0 | no |
|  | 1 | yes |
|  |  |  |

Codebook  
Codebook - Msvac - July 23, 2017

MsvacMsvac, table, 1 levels of column headers and 2 levels of row headers, table with 3 columns and 7 rows

|  |  |  |
| --- | --- | --- |
|  | | Value |
| Standard Attributes | Position | 54 |
|  | Label | Vaccination against Mycoplasma synoviae |
|  | Type | Numeric |
| Valid Values | 0 | no |
|  | 1 | yes |
|  |  |  |

Codebook  
Codebook - Erysipelasvac - July 23, 2017

ErysipelasvacErysipelasvac, table, 1 levels of column headers and 2 levels of row headers, table with 3 columns and 7 rows

|  |  |  |
| --- | --- | --- |
|  | | Value |
| Standard Attributes | Position | 55 |
|  | Label | Vaccination against Erysipelothrix rhusopathiae |
|  | Type | Numeric |
| Valid Values | 0 | no |
|  | 1 | yes |
|  |  |  |

Codebook  
Codebook - PDvac - July 23, 2017

PDvacPDvac, table, 1 levels of column headers and 2 levels of row headers, table with 3 columns and 7 rows

|  |  |  |
| --- | --- | --- |
|  | | Value |
| Standard Attributes | Position | 56 |
|  | Label | Vaccination against PD |
|  | Type | Numeric |
| Valid Values | 0 | no |
|  | 1 | yes |
|  |  |  |

Codebook  
Codebook - EDSvac - July 23, 2017

EDSvacEDSvac, table, 1 levels of column headers and 2 levels of row headers, table with 3 columns and 7 rows

|  |  |  |
| --- | --- | --- |
|  | | Value |
| Standard Attributes | Position | 57 |
|  | Label | Vaccination against EDS |
|  | Type | Numeric |
| Valid Values | 0 | no |
|  | 1 | yes |
|  |  |  |

Codebook  
Codebook - EDSvac\_inv - July 23, 2017

EDSvac\_invEDSvac\_inv, table, 1 levels of column headers and 2 levels of row headers, table with 3 columns and 7 rows

|  |  |  |
| --- | --- | --- |
|  | | Value |
| Standard Attributes | Position | 58 |
|  | Label | Vaccination EDS inverse |
|  | Type | Numeric |
| Valid Values | 0 | yes |
|  | 1 | no |
|  |  |  |

Codebook  
Codebook - ZRE\_1 - July 23, 2017

ZRE\_1ZRE\_1, table, 1 levels of column headers and 2 levels of row headers, table with 3 columns and 5 rows

|  |  |  |
| --- | --- | --- |
|  | | Value |
| Standard Attributes | Position | 61 |
|  | Label | Normalized residual |
|  | Type | Numeric |
|  |  |  |

Codebook  
Codebook - DFB0\_1 - July 23, 2017

DFB0\_1DFB0\_1, table, 1 levels of column headers and 2 levels of row headers, table with 3 columns and 5 rows

|  |  |  |
| --- | --- | --- |
|  | | Value |
| Standard Attributes | Position | 62 |
|  | Label | DFBETA for constant |
|  | Type | Numeric |
|  |  |  |

Codebook  
Codebook - DFB1\_1 - July 23, 2017

DFB1\_1DFB1\_1, table, 1 levels of column headers and 2 levels of row headers, table with 3 columns and 5 rows

|  |  |  |
| --- | --- | --- |
|  | | Value |
| Standard Attributes | Position | 63 |
|  | Label | DFBETA for Age in sampled house categories summarized(1) |
|  | Type | Numeric |
|  |  |  |

Codebook  
Codebook - DFB2\_1 - July 23, 2017

DFB2\_1DFB2\_1, table, 1 levels of column headers and 2 levels of row headers, table with 3 columns and 5 rows

|  |  |  |
| --- | --- | --- |
|  | | Value |
| Standard Attributes | Position | 64 |
|  | Label | DFBETA for Age in sampled house categories summarized(2) |
|  | Type | Numeric |
|  |  |  |

Codebook  
Codebook - DFB3\_1 - July 23, 2017

DFB3\_1DFB3\_1, table, 1 levels of column headers and 2 levels of row headers, table with 3 columns and 5 rows

|  |  |  |
| --- | --- | --- |
|  | | Value |
| Standard Attributes | Position | 65 |
|  | Label | DFBETA for Horses present inverse(1) |
|  | Type | Numeric |
|  |  |  |

Codebook  
Codebook - DFB4\_1 - July 23, 2017

DFB4\_1DFB4\_1, table, 1 levels of column headers and 2 levels of row headers, table with 3 columns and 5 rows

|  |  |  |
| --- | --- | --- |
|  | | Value |
| Standard Attributes | Position | 66 |
|  | Label | DFBETA for Use of litter inverse(1) |
|  | Type | Numeric |
|  |  |  |

Codebook  
Codebook - COO\_1 - July 23, 2017

COO\_1COO\_1, table, 1 levels of column headers and 2 levels of row headers, table with 3 columns and 5 rows

|  |  |  |
| --- | --- | --- |
|  | | Value |
| Standard Attributes | Position | 59 |
|  | Label | Analog of Cook's influence statistics |
|  | Type | Numeric |
|  |  |  |

Codebook  
Codebook - LEV\_1 - July 23, 2017

LEV\_1LEV\_1, table, 1 levels of column headers and 2 levels of row headers, table with 3 columns and 5 rows

|  |  |  |
| --- | --- | --- |
|  | | Value |
| Standard Attributes | Position | 60 |
|  | Label | Leverage value |
|  | Type | Numeric |
|  |  |  |

IBM SPSS Web Report

X

ABOUT

:   Created Using: IBM SPSS Statistics 23
:   Creation Date: Jul 23, 2017
:   Document Version: OriginalSaved Copy
:   Saved Date:  Jul 23, 2017

Navigation Controls

:   Contents - Opens and closes the list of charts and tables in the Web Report
:   Next & Previous - Display the next or previous table or chart in the Web Report
:   Help - Opens Help

Toolbar Buttons

|  |  |
| --- | --- |
|  | Undo - Undoes the last change in the document. |
|  | Edit - Open the Editor tool for tables and charts. Certain editing options are only available when you are connected to an Internet server. |
|  | Save - Creates a new copy of the Web Report with the saved changes. |
|  | Print - Prints the current object when in Object View and all objects in Page View. |
|  | Page View - Switches the Web Report to display all the tables and charts on a single page. |
|  | Object View - Switches the Web Report so that each table or chart is displayed one at a time. |

Connecting to a Server

:   The status of the Web Report's connection to an Internet server appears in the top right corner of the Web Report.
:   An Internet connection is not required to open a Web Report. With a saved copy of the Web Report you can view all of the charts and tables, and have some limited editing ability, when not connected to the Internet.
:   Connecting a Web Report to an Internet server will enable far greater editing capabilities for tables and for charts.

- If the author specified an Internet server when they created the Web Report, the Web Report will attempt to connect to the server automatically when it is opened.
- If the Web Report does not connect to a server, click on the server Status Message to open tools to retry the connection, try a different server, or enter a new server address.
- For information about adding the enhanced controls to your Internet Server, go to http://www.ibm.com/developerworks/spssdevcentral.
- If you specify a new server connection, the preferred format is http://xxx.xxx.xxx.xxx:xxxx.

Editing Tables

|  |  |
| --- | --- |
| Some of this functionality is only available when connected to an Internet server. | |
|  | Create a chart - Create a chart from the selected cells in the table. |
|  | Pivot and Sort - Transpose, sort, and pivot the table. |
|  | Background color - The background color of the selected cells. |
|  | Text Color and Style - Font color, style, and size. |
|  | Number Format - Font color, style, and size. |

Editing Charts

|  |  |
| --- | --- |
| All of this functionality is only available when connected to an Internet server. | |
|  | Chart Size - Change the height and width of the chart |
|  | Background color - The background color of the selected object. |
|  | Border and Line Style - The color and thickness of the line or border. |
|  | Text Color and Style - Font color, style, and size. |
|  | Number Format - Font color, style, and size. |
|  | Axis Properties - Change the scale and display axis titles and ticks. |

Save

X
New Name  
   
  
What to Save   
Save the entire document  
Only save the current object

Server Connection

X
  
Saved Server Connections  
   
  
  
  
Status

Add a chart

Pivot and Sort

Chart Size   
  

|  |  |  |
| --- | --- | --- |
|  |  |  |
|  |  |  |
| Lock aspect ratio | | |

Background   

|  |  |  |  |  |  |
| --- | --- | --- | --- | --- | --- |
|  | |  | |  | |
|  |  |  |  |  |  |
|  |  |  |  |  |  |
|  |  |  |  |  |  |

Line and Borders   

|  |  |  |  |  |  |
| --- | --- | --- | --- | --- | --- |
|  | |  | |  | |
|  |  |  |  |  |  |
|  |  |  |  |  |  |
|  |  |  |  |  |  |

Text Format   

|  |  |  |  |  |  |
| --- | --- | --- | --- | --- | --- |
|  | |  | |  | |
|  |  |  |  |  |  |
|  |  |  |  |  |  |
|  |  |  |  |  |  |

  

|  |  |  |
| --- | --- | --- |
|  |  |  |

  

|  |  |  |  |
| --- | --- | --- | --- |
|  |  |  | Agency FB Aharoni Algerian Andalus Angsana New AngsanaUPC Aparajita Arabic Typesetting Arial Arial Black Arial Narrow Arial Rounded MT Bold Arial Unicode MS Baskerville Old Face Batang BatangChe Bauhaus 93 Bell MT Berlin Sans FB Berlin Sans FB Demi Bernard MT Condensed Blackadder ITC Blood Of Dracula Bodoni MT Bodoni MT Black Bodoni MT Condensed Bodoni MT Poster Compressed Book Antiqua Bookman Old Style Bookshelf Symbol 7 Bradley Hand ITC Britannic Bold Broadway Browallia New BrowalliaUPC Brush Script MT Calibri Calibri Light Californian FB Calisto MT Cambria Cambria Math Candara Castellar Centaur Century Century Gothic Century Schoolbook Chiller Colonna MT Comic Sans MS Consolas Constantia Cooper Black Copperplate Gothic Bold Copperplate Gothic Light Corbel Cordia New CordiaUPC Courier New Curlz MT DaunPenh David DFKai-SB Dialog DialogInput DilleniaUPC DokChampa Dotum DotumChe Ebrima Edwardian Script ITC Elephant Engravers MT Eras Bold ITC Eras Demi ITC Eras Light ITC Eras Medium ITC Estrangelo Edessa EucrosiaUPC Euphemia FangSong Felix Titling Footlight MT Light Forte Franklin Gothic Book Franklin Gothic Demi Franklin Gothic Demi Cond Franklin Gothic Heavy Franklin Gothic Medium Franklin Gothic Medium Cond FrankRuehl Free 3 of 9 Free 3 of 9 Extended FreesiaUPC Freestyle Script French Script MT Gabriola Gadugi Garamond Gautami Georgia Gigi Gill Sans MT Gill Sans MT Condensed Gill Sans MT Ext Condensed Bold Gill Sans Ultra Bold Gill Sans Ultra Bold Condensed Gisha Gloucester MT Extra Condensed Goudy Old Style Goudy Stout Gulim GulimChe Gungsuh GungsuhChe Haettenschweiler Harlow Solid Italic Harrington High Tower Text HP Simplified HP Simplified Light IDAutomationHC39M Impact Imprint MT Shadow Informal Roman IrisUPC Iskoola Pota JasmineUPC Jokerman Juice ITC KaiTi Kalinga Kartika Khmer UI KodchiangUPC Kokila Kristen ITC Kunstler Script Lao UI Latha Leelawadee Levenim MT LilyUPC Lucida Bright Lucida Calligraphy Lucida Console Lucida Fax Lucida Handwriting Lucida Sans Lucida Sans Typewriter Lucida Sans Unicode Magneto Maiandra GD Malgun Gothic Mangal Marlett Matura MT Script Capitals Meiryo Meiryo UI Microsoft Himalaya Microsoft JhengHei Microsoft JhengHei UI Microsoft New Tai Lue Microsoft PhagsPa Microsoft Sans Serif Microsoft Tai Le Microsoft Uighur Microsoft YaHei Microsoft YaHei UI Microsoft Yi Baiti MingLiU MingLiU-ExtB MingLiU\_HKSCS MingLiU\_HKSCS-ExtB Miriam Miriam Fixed Mistral Modern No. 20 Mongolian Baiti Monospaced Monotype Corsiva MoolBoran MS Gothic MS Mincho MS Outlook MS PGothic MS PMincho MS Reference Sans Serif MS Reference Specialty MS UI Gothic MT Extra MV Boli Narkisim News Gothic Niagara Engraved Niagara Solid Nirmala UI NSimSun Nyala OCR A Extended Old English Text MT Onyx Open-Dyslexic Palace Script MT Palatino Linotype Papyrus Parchment Perpetua Perpetua Titling MT Plantagenet Cherokee Playbill PMingLiU PMingLiU-ExtB Poor Richard Pristina ProteinFontChem ProteinSpacefillBW Raavi Rage Italic Ravie Rockwell Rockwell Condensed Rockwell Extra Bold Rod Sakkal Majalla SansSerif Script MT Bold Segoe Print Segoe Script Segoe UI Segoe UI Emoji Segoe UI Light Segoe UI Semibold Segoe UI Semilight Segoe UI Symbol Serif Shonar Bangla Showcard Gothic Shruti SimHei Simplified Arabic Simplified Arabic Fixed SimSun SimSun-ExtB Snap ITC Spranq Eco Sans Stencil Sylfaen Symbol Tahoma Tempus Sans ITC Times New Roman Traditional Arabic Trebuchet MS Tunga Tw Cen MT Tw Cen MT Condensed Tw Cen MT Condensed Extra Bold Utsaah Vani Verdana Vijaya Viner Hand ITC Vivaldi Vladimir Script Vrinda Wageningen UR Logofont Webdings Wide Latin Wingdings Wingdings 2 Wingdings 3 |

Number Format   
  

|  |  |  |
| --- | --- | --- |
| 0.00 |  |  |

Axis Options   
  

|  |  |  |
| --- | --- | --- |
|  |  |  |
|  |  |  |
| Display Axis Title | | | |
| Display Ticks | | | |
